# Discussing parenthood with gay men diagnosed with HIV: a qualitative study of patient and healthcare practitioner perspectives

**DOI:** 10.1186/s12889-021-12285-4

**Published:** 2021-12-19

**Authors:** Robert Pralat, Jane Anderson, Fiona Burns, Elizabeth Yarrow, Tristan J. Barber

**Affiliations:** 1 Department of Sociology, University of Cambridge, 16 Mill Lane, Cambridge, CB2 1SB UK; 2 Homerton University Hospital NHS Foundation Trust, London, UK; 3 Institute for Global Health, University College London, London, UK; 4 Royal Free London NHS Foundation Trust, London, UK; 5 Centre for Gender Studies, University of Cambridge, Cambridge, UK

**Keywords:** Gay men, HIV diagnosis, Patient–provider communication, Reproductive health, Sexual health

## Abstract

**Background:**

Research on HIV and reproduction has focused largely on women and heterosexual men. This article examines whether it is relevant to address parenthood in HIV care with gay men and what ways of doing so are most appropriate.

**Methods:**

Qualitative interviews were conducted at four London clinics with 25 men living with HIV, aged 20–45, who did not have children, and 16 HIV clinicians. A thematic analysis identified potential reasons why parenthood was rarely discussed with gay men in HIV care.

**Results:**

Two sets of ideas contributed to a lack of conversations about parenthood: clinicians’ ideas about what matters to gay men and men’s ideas about what it means to be HIV-positive. Both sets of ideas largely excluded having children, with patients and practitioners similarly unlikely to raise the topic of parenthood in the clinic. Contrary to what clinician commonly assumed, many men expressed interest in receiving more information, highlighting the importance of reassuring people upon diagnosis that it is possible to become parents while living with HIV.

**Conclusions:**

Parenting desires and intentions were rarely discussed with men in HIV care. Our findings illuminate the potentially beneficial effects of emphasising that having children is a possibility at diagnosis, regardless of patients’ gender or sexuality. Conveying this information seems meaningful, not only to men who want to become parents in the future but also to others, as it appears to alleviate fears about mortality and ill health.

**Supplementary Information:**

The online version contains supplementary material available at 10.1186/s12889-021-12285-4.

## Background

There is now a substantial amount of research exploring reproductive desires, intentions and decision-making among people living with HIV, including studies from Europe [1–4], South Africa [5, 6] and the United States [7–10] that pertain specifically to men or incorporate men’s perspectives. Research in this area has focused on heterosexual men, but a small number of studies indicate that parenting desire is common also among gay, bisexual and other men who have sex with men (MSM) [11–13]. It remains unclear, however, whether healthcare practitioners discuss parenthood with their MSM patients and how relevant such discussion is to the HIV care of this patient group.

In many countries, such as the UK, increasing numbers of gay men are deciding to become parents. Whereas many gay men, especially from older generations, have children from previous heterosexual relationships, more recently there has been growing public visibility and social acceptance of men who pursue parenthood *after* coming out as gay, through routes such as adoption and surrogacy or by providing sperm to (and, in some cases, entering into co-parenting arrangements with) female friends and other women. In the UK, both partnered and single gay men have the legal right to pursue these routes to become parents, even if doing so can be logistically difficult or financially prohibitive.

While there is a striking lack of research on bisexual fathers [14], a large body of work documents experiences of parenthood among gay men, especially in Australia [e.g., 15–17] and the United States [e.g., 18–20], with most of the recent studies focusing on surrogacy. This research has provided much insight into why gay men decide to become parents, how they choose a specific route to parenthood and what kind of considerations these decisions involve. In studies of gay fathers, the men’s families are often situated among historical shifts in same-sex intimacy and kinship, including the advent of AIDS in the 1980s [e.g., 21–23]. But apart from a small number of memoirs written by HIV-positive gay fathers who have adopted [24], there is little discussion in the existing literature about the role HIV plays in gay men’s decisions to become parents or remain childfree.

There is little overlap between research on reproduction in the context of HIV and research on gay fatherhood. Gay *and* HIV-positive parenthood remains understudied, despite the fact that more gay men than ever before are living with HIV. Based on estimates from Public Health England [25], 1 in 11 gay and bisexual men in England – and 1 in 8 in London – are HIV-positive, with a total of 49,800 gay and bisexual men estimated to be living with HIV in the UK in 2018. While it is possible that few of these men decide to become parents, the lack of explicit attention to reproductive desires, intentions and decision-making among non-heterosexual men living with HIV is somewhat surprising when we consider, on the one hand, medical advancements that enable HIV-positive people to lead healthier and longer lives, and on the other, the increasing visibility and acceptance of gay fatherhood [26].

It is now well-documented that, although parenting desires and intentions are likely to be shaped by a variety of factors [27, 28], being HIV-positive does not in itself stop people from wanting or planning to have children [4, 5, 10]. The effect of antiretroviral therapy on HIV transmission has made parenthood a more accessible goal for both women and men living with HIV. There is now a consensus among clinicians that effective antiretroviral treatment, which lowers the viral load to levels that are ‘undetectable’, *eliminates* the risk of HIV transmission to sexual partners [29, 30]. As a result, at least in the UK, heterosexual couples where the man is HIV-positive have been increasingly advised to conceive ‘naturally’ [31]. This follows over a decade of treatment with a technique known as sperm washing which had previously allowed hundreds of HIV-positive men in Europe to conceive children without transmitting the virus to either the child or the mother [32].

Existing research suggests that issues around parenthood, conception and fertility are rarely discussed with men as part of HIV care. Studies in the UK [1] and the United States [9] found that few heterosexual men had discussed fatherhood with healthcare professionals, even though many men expressed the desire to have a child or had considered having children. Similar findings come from research with HIV-positive MSM. In a study of men attending a London HIV clinic, about one third of the MSM respondents reported having considered parenthood [13]. However, over three quarters reported not having had any discussion with a healthcare professional about the possibility of becoming a parent, while over two thirds indicated that they were insufficiently informed. A more recent study conducted in the United States found that similar proportions of women, heterosexual men and MSM expressed parenting desire, concluding that, regardless of gender or sexual identity, people living with HIV ‘commonly desire children at rates similar to their HIV-negative counterparts and with higher frequency than in the early HIV era’ [11 , p. 6]. This is reflected in findings from a recent study of serodiscordant relationships (with one partner HIV-positive and the other HIV-negative) in Australia, where some HIV-positive gay men and their partners pursued, or considered pursuing, various routes to parenthood, including surrogacy and fostering [12].

As existing evidence shows, some non-heterosexual men living with HIV have children and many would like to become parents in the future. However, it seems that parenthood is rarely discussed with men in the context of HIV care, leaving them potentially uninformed about the implications of being HIV-positive for considerations about having children. The only study that examined this issue specifically in relation to MSM [13] was conducted in the early 2000s when both the awareness of gay fatherhood and the possibilities to have children were more limited than they are now. While more recent research [12] identifies parenthood as an issue that is increasingly relevant to gay men living with HIV, there remains limited understanding of the needs and challenges that men who are non-heterosexual and HIV-positive are likely to face as they consider becoming parents in the future. There is also, to the best of our knowledge, no previous research that has explored this issue from the perspective of HIV clinicians. To address this gap in the literature, we designed a qualitative study attending to both patient and practitioner perspectives. If, as findings of existing research suggest [1, 9, 13], HIV clinicians are unlikely to discuss parenthood with men, it is important to understand why this may be and if any pertinent questions are left unanswered.

## Methods

Between May and December 2016, the first author conducted qualitative semi-structured interviews with healthcare practitioners (*n* = 16) and patients (*n* = 25) at four HIV clinics in London. The clinics were selected due to their different patient demographics and the fact that they were in different areas of London, characterised by contrasting demographics of the local populations (including more affluent and relatively deprived areas).

As we report elsewhere [33], we sought to interview men living with HIV who were gay or bisexual, 20–45 years old and without children, as well as clinical staff working with this patient group. In order to capture a range of practitioner perspectives, we decided to recruit staff in four main clinical roles: nurses, physicians, psychologists and sexual health advisers. To attract a wider pool of patients – not only those interested in having children – we advertised the study as research on men’s attitudes to intimate life, including sexuality and fertility. This was to help us understand how the possibility of becoming a parent relates to other aspects of personal relationships. Recruitment for the study was facilitated by local clinical research teams who were advised that it was important for us to reach men with a variety of views about parenthood as well as different cultural backgrounds.

### Participants

Healthcare practitioner interviewees included five physicians, five sexual health advisers, three nurses and three psychologists; ten women and six men. For some, more than 90% of their patients were MSM; for others, less than a half. Many practitioners had previously worked in clinics with different patient demographics. Some had worked in HIV medicine since the 1980s; others had begun working in this area more recently.

Table 1 shows demographic characteristics of patient interviewees. The men were aged between 20 and 45 (the median age was 35); they were born between 1970 and 1995. Their age at HIV diagnosis ranged from 20 to 34 (the median age was 29) and the time since diagnosis from one month to 15 years (the men were diagnosed between 2001 and 2016). All men were on antiretroviral treatment at the time of the interview. Although they were not asked about it directly, most men mentioned at some point during the interview that they were ‘undetectable’ and none indicated that they were not.

**Table 1:** Demographic characteristics of study participants (men living with HIV)

Participant characteristic		n
Age (in years)	20–29	8
	30–39	13
	40–45	4
Sexual identity/orientation (self-identified)	Gay	23
	Bisexual	1
	Other	1
Country of birth	UK	13
	Other country in Europe	4
	Country outside Europe	8
Ethnicity	White	17
	Asian	5
	Mixed-race (white/black)	2
	Black	1
Education	University degree completed	22
	University degree in progress	1
	Other	2
Employment	Employed (full- or part-time)	22
	Unemployed	2
	In education	1
Partner	HIV-negative man	8
	HIV-positive man	3
	N/A – single/dating	14

### Interviews

Interview materials, including participant information sheets, consent forms and topic guides (with indicative questions), were consulted with two patient champions based at one of the clinics and with UK-CAB, an HIV treatment advocates network. The topic guides are included as supplementary information (see additional files 1 and 2). Interviews took place in private rooms in the clinics where participants had been recruited and were audio-recorded.

Interviews with HIV clinicians covered contexts in which parenthood or reproductive health was addressed in their work with patients, their experiences of discussing reproduction with MSM and their perceptions of how MSM’s intimate relationships had changed over time. The average length of practitioner interview recordings was just under an hour.

Interviews with men living with HIV covered their feelings about parenthood, experiences of intimate and personal relationships, and views about different ways of having children. HIV was addressed in relation to these topics based on information interviewees volunteered, usually unprompted. Towards the end of the interview, the men were asked if they had ever discussed parenthood or reproductive health with HIV clinicians, whether they would like, or would have liked, to discuss this topic, and whether they thought there were any needs for support or information. The average length of patient interview recordings was just over an hour and a half. All patient interviewees received a £25 gift voucher as a thank you for their time.

### Data analysis

Once the interviews had been transcribed, the first author anonymised and analysed the transcripts. Each transcript was read multiple times in search of common themes. For the purpose of analysis presented in this article, data that concerned both HIV and parenthood were analysed in greater detail. A number of themes captured sentiments expressed in multiple interviews. Some of the themes related to communication, conversations and interactions between patients and practitioners, and issues pertaining to discussing parenthood as part of HIV care became the primary focus for the article. The subsequent analysis concentrated on relevant interview extracts and it was guided by specific questions identified in existing literature. Having analysed the data, the first author drafted the article to which other authors contributed.

Our approach to analysis was both deductive and inductive. We had a specific interest in patient–provider communication and in hearing about conversations that HIV clinicians and men living with HIV had (or had not) experienced when providing or receiving HIV care. At the same time, the analysis was data-driven and it was through the close reading of interview transcripts that concepts, categories and distinctions that inform our findings were identified. Our analysis was attentive to overlaps and contrasts between patients’ and practitioners’ accounts. As such, we consider the two sets of interviews as offering complementary perspectives on communicating about parenthood in HIV care.

### Terminology

Throughout the article, when describing the group of men who are the focus of our study with respect to their sexuality (sexual identity or orientation), we tend to use the term ‘gay men’. Although our original intention was to capture views and experiences of both gay and bisexual men, only one man who identified as bisexual took part in our study, which meant that we were not able to represent bisexual perspectives in a meaningful way. Since the vast majority of men identified as gay, we adopt this term to describe the men as a collective, while remaining attentive to potential relevance of our findings to bisexual men and to the accounts of patient interviewees who did not identify as gay. In order to reflect terminology used by practitioner interviewees (and in much of the literature we draw upon), we also use the term ‘MSM’ when describing men as a patient group.

We use the terms ‘parenthood’ and ‘having children’ interchangeably and in a broad sense, encompassing various possible meanings of being a parent. While we are not limited in our focus to ‘biological’ parenthood, we recognise that, by taking part in our study, interviewees were invited to talk about having children *in relation to* living with HIV. Perhaps inevitably, interviews ended up focusing to a large extent on biological parenthood, which did not necessarily reflect the men’s interests in pursuing it.

## Results

In our interviews, both HIV clinicians and men living with HIV confirmed what would be expected based on existing research: discussing parenthood with gay men in HIV care was not common. In our analysis, we focused on *why* this was the case and whether, from the perspective of patients or practitioners, there was a need for more communication. In what follows, we show how two sets of ideas contributed to the absence of conversations about parenthood: clinicians’ ideas about *what matters to gay men* and men’s ideas about *what it means to live with HIV*. We found that both sets of ideas largely excluded having children, which meant that neither patients nor practitioners were likely to initiate such conversations in the clinic. We present our findings in two sections in which we focus on the interplay between what is *assumed* and what is seen as *possible*, and on the distinction between receiving *healthcare* and receiving *a diagnosis*. Throughout our analysis, we refer to practitioner interviewees by specifying their profession, and to patient interviewees using pseudonyms and indicating their age.

### What matters to gay men: limited conversations and unspoken assumptions

Practitioners who took part in our study knew if their patients had children and most of them had a small number of fathers among their MSM patients. Most common were older men with children from previous heterosexual relationships who came out as gay later in life. Some practitioners mentioned younger men, usually from ethnic minorities, who had children with female partners, while, at the same time, having sexual relationships with other men. Also mentioned were individual cases of gay men who had pursued surrogacy or adoption. As one physician noted, asking new patients whether they already had children was crucial ‘because you need to make sure that any children who are at risk are also tested for HIV’. But this initial question was where conversations about parenthood usually ended. Another physician explained:

I do ask everybody if they have kids. Because even if they are, you know, a 20-year-old MSM, you don’t know what happened in their teens. So I always ask if they have kids. And sometimes they’re like, oh god no! And you’re like, okay, well, I don’t need to have that conversation.

A sexual health adviser described how MSM patients were sometimes baffled when they were asked if they had children: ‘I’ve seen older guys who have looked at me like I was literally from outer space – like, what are you talking about? As in it’s never occurred to them, it’s just so impossible.’ Based on practitioners’ accounts, it was standard practice to ask new patients, including MSM, if they *had* children. However, questions about *parenting desires or intentions* were rarely directed at gay men. As one physician noted, ‘I can’t think of any gay men with whom I’ve had a conversation about planning to have kids. And I don’t know whether that’s because they’re not planning to or just because I haven’t asked them. We haven’t had those conversations.’

Not having ‘those conversations’ meant that *reasons* for not having them remained unclear. Some practitioners recognised that just because a particular issue was not mentioned by patients did not mean that it was of no interest. At the same time, they were conscious about not ‘pushing an agenda’ by addressing issues that patients did not raise themselves. Reflecting about male patients more broadly, a psychologist pondered:

I haven’t had that much of an opportunity to have that conversation with men. And I think because, you know, we are patient-led in the work that we do, I would never presume something unless it’s raised with me… So if somebody’s referred to me for something and we’re working on it, I feel I would be presumptuous to say, oh, and have you thought about having kids? But then, again, might there be reasons why men haven’t necessarily been raising this issue with me?

Sometimes practitioners’ personal circumstances made it more likely for conversations about parenthood to come up. As one nurse recalled, ‘I might have said in just general chit chat – oh, do you want kids? And I think it’s something that came up with patients when I was pregnant.’ A physician similarly acknowledged: ‘My daughter is adopted and a lot of [my patients] know that. So I think that kind of makes it easier for them to talk about [parenthood] in a way.’

The comments from practitioners highlight the interactive and relational nature of HIV care. They also convey a friendly and informal character of the patient-provider relationship, which both HIV clinicians and men living with HIV often remarked upon. It was evident from both sets of interviews that clinical interactions in HIV care were rarely just about health: it was not unusual for patients and practitioners alike to be knowledgeable about each other’s personal circumstances. Indeed, some clinicians were surprised how little they knew about their patients’ views about parenthood considering how familiar they were with other aspects of the men’s private lives. A sexual health adviser contemplated:

I do think I’m used to talking to gay guys about sex and intimacy and the things which get in the way of that, and all the painful feelings which sometimes could be brought up, and helping them work through those feelings in terms of, you know, having enjoyable sex and an enjoyable sex life. But I haven’t… I think there was something within me which wasn’t allowing the possibility that an HIV-positive guy could be a… dad. And that’s bonkers.

Some practitioners noted that they would not ask gay men about parenthood, even if they asked other patients about it. As one physician explained:

I suppose I don’t seek such conversations with many of my patients, I wouldn’t ask them that. You know, I’d respond if I was asked, but I wouldn’t give them information, perhaps in the way I would if it was with a straight man or a woman. Which may be wrong.

Similarly, a nurse observed:

I would automatically ask women about fertility. I’d probably ask heterosexual men if they’re in a relationship – you know, have you got plans to have children? And I suppose because it’s a different mechanical process for MSM and how you go about that… potentially I am doing a disservice because I’m not asking about it.

These two quotations show how practitioners questioned their approach, openly reflecting on their clinical practice during the interviews: they acknowledged that not asking MSM about parenting intentions ‘may be wrong’ – potentially, it can constitute a ‘disservice’. The clinicians also recognised the role that gender and sexuality played in their interactions with patients: whereas women would ‘automatically’ be asked about plans to have children, gay men were unlikely to be asked a similar question. Another nurse reflected at length on how gender and sexuality shaped assumptions about what was important to different kinds of patients:

No matter how open-minded clinicians feel, I think that if you’ve got a woman of a certain age who’s heterosexual in front of you, there’s sort of an automatic thing the doctors will say – oh, you can still have children! There’s something about making that assumption – that that’s important to that person because of her gender. And because of her age. I can’t imagine, I don’t know if [others] do that, but my experience of the people that come to me is that’s not done with men – probably not even that much with straight men either, I don’t know. I think it might be more of a gender thing than it is a sexuality thing. And the difference, I think, is that the men probably have to take an active role in asking. Whereas my experience with women is it tends to be kind of thrown at them. And it’s not that, I wouldn’t say that people working here are particularly prejudiced – they’re not. I think it’s just the assumptions that we make about, you know, certain people – we think, okay, they might want children, so I’m going to reassure them that’s okay. But we wouldn’t necessarily do it to everybody that we meet.

This quotation sheds light on how clinical practice can be guided by assumptions made on the basis of patients’ gender and (perceived) sexual identity. Clinicians make assumptions about their patients not necessarily because they are ‘prejudiced’ or insufficiently ‘open-minded’ – they can assume that certain things matter because they want to be reassuring about what they consider to be important to the patient. This affirming position echoes the earlier account from the sexual health adviser who, in addition to ensuring that her patients took care of their health, helped them achieve ‘an enjoyable sex life’. However, this seemingly non-judgemental approach is not exactly free of judgement: even if clinicians recognise that, in general terms, having sex is not less important or valuable than having children, their practice can still reproduce stereotypes along gender and sexual lines.

A number of practitioners commented how participating in the study made them more aware of potential barriers to addressing the issue of parenthood with gay men. One sexual health adviser observed:

I suppose this whole [interview] has made me reflect on my practice and what happens here and what doesn’t get talked about and why that may be. So I suppose it’s been a thoughtful process for me in that sense. Yes, maybe there’s a lot of unspoken assumptions. I suppose we’ve probably moved on from the idea of gay men don’t have children. But being able to move further forward in terms of talking about it more… maybe there’s still kind of stumbling blocks around there.

As this quotation highlights, absorbing the fact that it is possible for gay men to be fathers can be a gradual process. For this clinician, the notion of gay fatherhood might have become more *thinkable* (‘we’ve probably moved on from the idea of gay men don’t have children’), but moving ‘further forward’ and being adept at *talking about it* can take some time.

Interviews with men living with HIV echoed interviews with HIV clinicians in that they also showed that conversations about parenthood in HIV care were uncommon. Of the 25 men interviewed, only four could recall discussing the possibility of having children with clinicians. Two men had been told that despite being HIV-positive they were still able to become parents and two other men had asked if this was an option. Interestingly, both men who did recall being told that having children was a possibility received care at the only clinic where the majority of patients were women. The two men who asked about parenthood themselves, both South/Southeast Asian, raised the topic at different points: one at the time of being diagnosed with HIV and the other one when he had already been on HIV treatment. The man who inquired about parenthood when he received the diagnosis was also one of the two men who did not identify as gay. He said: ‘That was the first question I asked them – will I be able to have children?’ The other man, who wanted to adopt a child with his partner, had asked his HIV consultant if it was an option and had been told that ‘it should be alright’.

Some men were not sure if they had ever discussed parenthood with HIV clinicians, emphasising the amount of information they had to grapple with following the diagnosis:

It’s hard to know entirely because I’ve, A, been told so much and, B, searched so much, so the two kind of cross over. It’s something which I’m almost certain has been mentioned to me in no great depth whatsoever. (Blake, mid-20s)

I must admit there have been a lot of things and places and professionals and blah, blah, blah, that it’s so hard to… I just lose track, even of dates and stuff… But I do not remember really, and I don’t think so. (Juan, early 40s)

Other men noted that taking part in the study was their first opportunity to talk about parenthood in the context of HIV. For example, Lee, in his late 20s, said: ‘You were the first encounter I’ve had in relation to my health – like, HIV – and children.’ Ben, in his mid-30s, made a similar comment: ‘Other than the discussion with you, I don’t think that it has ever been discussed with me before.’

The men who took part in our study expressed a range of parenting desires, which had been shaped in complex ways. Based on how they reflected on their feelings about parenthood during the interview, 12 men could be described as wanting to become parents in the future, nine did not want to have children and four were undecided or could not be placed in either category. Parenting desire (or lack thereof) seemed somewhat dependent on age: for example, none of the eight men in their 20s said that they *did not want* to become parents at some point, though none of them *intended* to do so any time soon. In contrast, perhaps surprisingly, feelings about parenthood did not seem to be influenced by the men’s partnership status. Few men actively planned to become parents and, for most men, having children was not a priority. However, irrespective of their reported feelings about parenthood, many men highlighted that, to various degrees, it would be useful to discuss parenthood as part of HIV care:

I think that, even though I don’t have any intention, you know, [to become a parent], it’s something I’d be curious about – in case, you know, I decide to do it. (Juan, early 40s)

I don’t think there would be a need for that much detail unless someone asked for it. But if someone were to sit you down and go, here are your fertility options, it would be very useful – just to know that that door is still open. (Lewis, early 30s)

I think I had a lot of questions that came out of [this interview] that I didn’t realise were there. So I think that alone is justification for there being some kind of provision for discussing parenting. (Lee, late 20s)

Similar to the sexual health adviser who noted how taking part in the study made her ‘reflect on my practice’, Lee (quoted above) remarked how participating in the interview made him aware that he ‘had a lot of questions’ which he ‘didn’t realise were there’. Such reflections demonstrate how the limited *conversations* in the clinic can be both a cause and an effect of a limited *consciousness* about what is possible. As one clinician quoted earlier observed, ‘there was something within me which wasn’t allowing for the *possibility* that an HIV-positive guy could be a… dad’. Having children was not part of the conversation, because parenthood in this context was not thinkable – it was not *imaginable*. A similar constraint of the imagination was evident in men’s accounts about their understandings of living with HIV.

### What it means to live with HIV: constrained futures and unrealised possibilities

So far, we have seen how rare it was for the men and clinicians to talk with one another about the possibility of having children. We have suggested that the lack of conversations about potential parenting desires or intentions could be partly explained by what clinicians assumed about what mattered to their patients. Assumptions aside, sometimes practitioners had good reasons for not asking men about parenthood – if a man looked at them as if they were ‘from outer space’, it was understandable to conclude that they ‘didn’t need to have that conversation’. Some men also suggested that it was not necessary for clinicians to initiate such conversations with their patients:

If a person is actually wanting to explore that fatherhood avenue, I think then that person should be really wanting to open that avenue with their consultant. I don’t really think it should be a rule for every gay man that comes into the clinic. But you could put some signs out that say, well, if there ever is such a need then you know who to actually speak to. (Tony, late 30s)

[Clinicians] don’t have the luxury of time to give that much information… So I don’t think that this needs to be sort of included in the healthcare, you know, like part of your routine check-up. I think what should be provided is a little bit of, look, if you have questions about this, you can search this webpage. (Lucas, early 40s)

As these two quotations show, even though some men did not see a need for clinicians to proactively discuss parenthood with patients, they nevertheless suggested that information about having children could be communicated in other ways and that opportunities to receive such information could be more transparent. One of the key themes in the interviews was the importance of being reassured that having children was an option for people living with HIV. Whether or not the men wanted to become parents in the future, they highlighted the significance of this reassurance. They also overwhelmingly suggested that it was best, and easiest, to convey this message at the time of the diagnosis. One man, Rory, in his mid-30s, commented:

I just think [having children] is not something that can be addressed in a sort of, like, you know, your update appointment… Maybe it could just become part of a, you know, sort of general diagnosis, just part of that general checklist of things – like, you know, these are the things you need to be aware of, these are the things you need to look after. Oh, and, you know, if you’re considering having a family or, you know, want to have kids, then there is… there are options.

Many men identified the HIV diagnosis as the most appropriate context for raising the issue of parenthood. They seemed to agree that it was important for clinicians to highlight, if this was true, that being HIV-positive should not in itself prevent people from having children. This was regarded as a valuable – and usually sufficient – message, which the men would have appreciated (or did appreciate) receiving at the time of being told about their HIV status. Ben, in his mid-30s, tried to remember what information was conveyed to him during his HIV diagnosis:

I could be wrong but I don’t recall being diagnosed and then being asked, does this bother you about parenthood? I don’t think that’s ever been discussed with me. And I do think that it should be discussed with those that are interested. I don’t know if it’s even possible for me to have a child, because I don’t know if I’m going to transmit HIV to the child or to the mother. So even if that was just very briefly explained to me – that it is possible to have a child and not have them be HIV-positive, so if you ever did want children, you can go about seeking it, and we’ve got this support group or this organisation that you can go to…

Unsure ‘if it’s even possible for me to have a child’, Ben emphasised his limited knowledge about HIV transmission. The incomplete understanding of how HIV is passed on was another common theme in patient interviews, as we discuss in detail elsewhere [33]. Another man, Lewis, in his early 30s, shared his memories from the time he was diagnosed, explaining what kind of information he wished he had received: ‘At the time [I was diagnosed], I thought, well, long-term partnerships are done – you know, nobody will want to be with me, unless it’s out of pity. Having children – well, whatever ideas I might have had, that’s done now, that’s not going to happen.’ Even though, for Lewis, receiving an HIV diagnosis initially meant that both long-term partnerships and having children were ‘done’, he explained later in the interview how he was ultimately reassured about partnering but not about parenting:

I think if you’ve just been told at the beginning of your journey, as it were, that [having children] is a possibility and, if you do want to talk to us about it, we can put you in contact with people – for me personally that would be enough. Just to go, okay, cool, it’s an option, you know, it’s not impossible… Because having a child is a very practical thing, in a sense. So if that had been explained to me, that actually, in a practical sense, it is possible, here are your options – so you’re positive now, what next, you know… They told me, listen, you’ll have boyfriends, you’ll be fine, blah, blah, blah. But at that point, you can’t really hear that… The idea of having kids… just in a practical sense, like, well, this cannot happen. You know, my sperm is now sullied, it cannot be used by anyone.

Lewis made a distinction between having boyfriends and having children, highlighting how the latter was ‘a very practical thing’. The conversation he remembered from the time he was diagnosed with HIV was in many ways reassuring. However, the expanded understanding of what it meant to be HIV-positive did not incorporate the practicalities of becoming a (biological) parent, which meant that the prospect of having children seemed unattainable. Mike, in his mid-30s, recalled feeling similarly resigned when he was told about his HIV status:

Before I was diagnosed, [a family with children] – that’s kind of what I wanted to have, you know. And since being diagnosed I’ve kind of… it doesn’t even cross my mind anymore… It’s not something that I’ve been very kind of traumatised by. But I guess before I was diagnosed I had always grown up thinking, you know, just because I’m gay doesn’t mean that I will never have children… But since the diagnosis I’ve just kind of thought, well, that’s just not going to be possible now.

It is telling that Mike talked about being gay, which some could regard as a more significant barrier to parenthood, as much less of an obstacle to having children than being HIV-positive. He elaborated later in the interview:

I guess [when I was diagnosed] I just thought, well, that means that if I wanted to have a child it would mean that child would have HIV. And I guess I don’t really understand it that much. I don’t understand how people get around that – or if you can even get around that… But in my mind, I had kind of made up my mind that it’s just something that wouldn’t be possible.

Similar to Ben quoted earlier, Mike was highly concerned about the risk of passing HIV on to the child, stressing his lack of understanding of HIV transmission. But not having this knowledge did not make him seek it. Instead, he had *made up his mind* that he would not be a parent in the future – ‘a family with children’ was a possibility that ‘did not even cross his mind anymore’.

Feelings of resignation that being diagnosed with HIV can evoke, expressed by men such as Mike, were also described by some practitioners. A psychologist recalled:

I had a patient and the one thing that he found most upsetting when he was diagnosed was – you know, he could deal with all the health stuff and he knew a lot about, you know, if I’m on my meds I’ll be fine, you know, it’s a long-term path, he knew that kind of narrative – but what he hadn’t realised is that it may be possible for him to have children. He was a gay man and he was likely to probably have children with a straight female friend who wanted to have a child but didn’t have a partner… And he just assumed that it would no longer be possible… And I said, well, it’s not impossible – you know, there are ways in which these things can be done… And he sort of burst into tears and was like, oh, I had no idea that, you know, that that could happen.

The psychologist highlighted how much impact an HIV diagnosis can have and how much of a relief it can be for patients to have their assumptions immediately challenged:

A new diagnosis is often – this is over now. And then there’s the relief when you say, well, it can still happen, it just… you know, you just have to think about it in a different way and, you know, there are ways and we can talk about that later – I don’t tend to go into detail about things like that at that time. But it’s about sort of saying, you know, those avenues are not completely shut.

Recognising that, upon diagnosis, patients may not have the mental capacity to ask about things that matter to them led some practitioners to adopt a more proactive approach. As one physician explained:

[People who are newly diagnosed] have so much stuff going on that sometimes I think it’s just good to say it for them, you know. They’re thinking about lots of things and then sometimes I think they’re a little bit overwhelmed… You just try to demonstrate for them that they still have all the options that they would have had otherwise. Those options might just take a little bit more of a workaround.

As we can see from both patient and practitioner accounts, an HIV diagnosis is often overwhelming. The perceived seriousness of the diagnosis – the initial reaction that ‘this is over now’ – is underlined by the visible relief that follows when the patient’s attention is directed to possibilities that, in this very moment, are beyond his awareness. The significance of this shift in consciousness was emphasised by men living with HIV as well as HIV clinicians. For example, Ian, in his early 40s, for whom parenthood ‘was never really a big consideration’, spelt out how knowing that being HIV-positive does not preclude parenthood had implications beyond having children:

It’s that whole thing when you’re diagnosed – you do question the future… Even if I wasn’t interested [in having children], the very fact that someone could come and tell you, well, you can still be a parent, means that you actually then think, oh, well, then I can live much longer if you think I can be a parent – do you know what I mean? The two come hand in hand.

Ian’s comment illustrates why being told that ‘you can still be a parent’ is meaningful not only with regard to parenthood – it links to other issues such as longevity. Saying that having children is an option can have a vital effect, even on people with no desire to have children. Thinking about his work with newly diagnosed patients, a sexual health adviser explained how proactively raising the issue of parenthood made patients more future-oriented:

Throwing that into the conversation – you know, these are things that could happen in your future – that is something that allows [patients] to… focus ahead as opposed to here and now. So it’s almost like throwing that in is an opportunity to explain more about how the virus works and how it can be managed. It gives possibilities for sharing more information. It’s almost like you’ve sown a seed that instantly germinates. Because they will kind of respond, is that possible? And that is another opportunity for education.

As this quotation elucidates, focusing ‘ahead as opposed to here and now’ shifts attention towards possibilities that are unlikely to be evident at the time one receives an HIV diagnosis. The sexual health adviser presents the possibility of having children as ‘an opportunity’ – not necessarily to start thinking about parenthood, but to better understand ‘how the virus works and how it can be managed’. Crucially, ‘sharing more information’ can improve not only knowledge but also wellbeing. As one of the patients interviewed highlighted, knowing that he could still become a parent had positive effects on his mental health. Recalling his previous suicidal attempts, and having thoughts which he described as ‘a very dark side’, he said that being told he was able to have children when he was diagnosed could have been ‘one of the facts that made me want to, you know, not go to the dark side. It was that hope that, yes, it’s not the end, everything’s possible.’

In sum, our interview data illuminate how an HIV diagnosis can make some people assume that they can no longer become parents and how being told that this is not the case can evoke feelings of relief. Moreover, realising that parenthood is a possibility can mean more than recognising that one can have children – it can also shape perceptions of other issues, such as life expectancy. Consequently, being reassured that becoming a parent is an option matters not only to men for whom having children is important but also to others, as such reassurance seems to reinforce a more optimistic outlook on oneself and the future.

## Discussion

Based on interviews with patients and healthcare practitioners in London HIV clinics, we have found that the possibility of having children was rarely discussed with gay men as part of HIV care. Few practitioners reported talking about parenthood with their MSM patients and only a small number of men could recall discussing it with their HIV clinicians. This is perhaps not surprising, considering the common perception of reproduction, in clinical medicine and beyond, ‘as though it is something that happens only to women, matters only to women, and affects only women’ [34 , p. 437]. While there is a growing body of research on men’s perspectives on parenthood, especially in relation to infertility [e.g., 35–39], the focus on men’s reproductive health is relatively recent [40]. Especially in the context of HIV/AIDS, ‘men have been studied as sexual creatures, while women continue to be framed in reproductive terms’ [41 , p. 5]. Our study is one attempt to redress this imbalance.

In our study, the lack of conversations about parenthood (and, more specifically, about parenting desires and intentions) made some practitioners assume that having children was of little interest to gay men, while some patients questioned if it was even possible for HIV-positive men to become parents. Taking part in the study made some clinicians rethink their assumptions and it made some men realise that they would like to know more about what living with HIV meant in relation to parenthood. The accounts of both HIV clinicians and men living with HIV suggest that it is receiving a diagnosis, rather than healthcare, when the need to communicate about parenthood is most explicit. It also seems that introducing the topic of parenthood into a conversation can feel more natural, and potentially less awkward, when providing the diagnosis than when seeing existing patients as part of routine medical appointments. Our interviews revealed that the HIV diagnosis was a critical moment to communicate that *it was possible* for people living with HIV to have children. Furthermore, communicating this information seemed important not only to those interested in parenthood but to other men too, as it helped alleviate fears about mortality and ill health.

Our data show that, for many men, being diagnosed with HIV can lead to an internalised assumption that having children is no longer an option or that it is impossible to prevent HIV transmission to a child conceived with sperm from a man who is HIV-positive. This echoes findings from previous research with heterosexual men living with HIV in the United States, which shows that the belief that transmission is inevitable is a common misconception [8, 10].^1^ Whereas for some men in our study assuming that they could not become parents because of their HIV status was relatively harmless, as they were not interested in having children anyway, for others it was a damaging assumption as it spoilt the prospect of parenthood, which the men seemed to have cherished prior to their HIV diagnosis. Regardless of men’s feelings about having children, excluding parenthood as a possibility *because of HIV* seemed to contribute to negative perceptions of oneself or the future. In contrast, knowing that ‘that door is still open’ or that ‘those avenues are not completely shut’ appeared to foster more positive attitudes towards living with HIV.

Our findings contribute to previous research with gay men diagnosed with HIV, which has shown how receiving the diagnosis can be ‘both unsettling and confusing’, and how it can lead to ‘unwelcome and problematic changes in identity’ [42 , p. 1381]. Drawing on their earlier work, Flowers and colleagues suggest that, with the advent of effective antiretroviral therapy, being diagnosed with HIV changed from a ‘death sentence’ to a ‘life sentence’, and while the distress concerning life expectancy diminished, the psychosocial factors associated with the diagnosis remained or took on new meanings [42]. Whereas previous research on gay men diagnosed with HIV does not mention concerns about not being able to have children, at a time when parenthood has become more thinkable and imaginable for gay men [43], such concerns might become more prevalent. It is therefore important for HIV clinicians not to assume that having children is irrelevant to MSM patients and instead reassure them that it is indeed (still) an option.

Our findings highlight the potentially beneficial effects of including information about parenthood at diagnosis, but it is important to consider *how* to incorporate this information into conversations with patients. In the context of a serious health diagnosis, communication needs to be selective in order to avoid information overload. Previous research with gay men living with HIV has shown that information received at diagnosis can be difficult to absorb and that learning about being HIV-positive is ‘not a single clinical event’ but rather ‘a process of discovery that is experienced differentially, and often over a period of time’ [44 , p. 213]. Therefore, it seems that the possibility of having children should be communicated in simple terms, with more detailed information readily available to those who are interested in finding out more about the consequences of being HIV-positive for considerations about parenthood. Based on our findings, it is good practice for healthcare practitioners who are in charge of telling patients that they are HIV-positive to reassure them that having children remains a possibility, without assuming that this information is relevant only to some patients, because of their gender or sexuality. Moreover, it seems important for practitioners, if they are asked for further information, to be able to explain the practicalities of pursuing parenthood through means that HIV-positive gay men might consider (including adoption, surrogacy and sperm donation) and to direct patients to relevant resources.

In their research with African American women living with HIV/AIDS, Watkins-Hayes and colleagues argue that how one copes with an HIV diagnosis is shaped by how the diagnosis is framed: people newly diagnosed with HIV adopt positive or negative behaviours and attitudes depending on the *initial information* they are given about their HIV status, the *conceptual framework* they are offered to understand living with HIV, the *language* used to talk to them about HIV and the *tangible resources* they are provided with [45]. In turn, how HIV is framed affects people’s experience of HIV stigma, including the extent to which they internalise it by, for example, perceiving themselves as infectious [46–48]. Much of the information provided to patients upon diagnosis focuses on risk – or, increasingly, lack thereof. Our findings highlight the need to communicate in HIV-related healthcare interaction not only about risk but also about possibilities [49]. It is equally important to extend this framing beyond conversations with people newly diagnosed with HIV to how we communicate about HIV more generally in society at large.

This is, to the best of our knowledge, the first qualitative study to focus specifically on views about parenthood among gay men living with HIV, as well as the first study to examine discussions about parenthood in HIV care based on perspectives of both patients and healthcare practitioners. Unfortunately, despite the original aim to interview both gay and bisexual men, we did not succeed at recruiting a sufficient number of bisexual men to be able to represent their experiences meaningfully. One factor that might have contributed to the difficulty in recruiting bisexual men is the fact that potential patient interviewees were approached based on their clinical categorisation as MSM. Many bisexual men (especially those in relationships with women) might not have been categorised as such in the first place. It is worth noting that, in our interviews, some HIV clinicians said that they were not aware of any bisexual men among their patients; others seemed to use the term ‘MSM’ as synonymous with ‘gay’. This is an issue that is worth considering in future research which depends on recruitment of non-heterosexual men in clinics.

In addition to specifically attending to experiences of bisexual men, further research should examine the prevalence of different views and perspectives of men whose views are underrepresented in our study, notably black men, men in their early 20s and men without university education. It is noteworthy that the only two men in our study who had asked their HIV clinicians about parenthood were both South/Southeast Asian. This suggests that ethnicity, and related cultural expectations, might play an important role in shaping men’s views about parenthood, and is consistent with previous research [11, 39, 50]. Indeed, our study, with its predominantly white group of interviewees, may underestimate the importance of parenthood among HIV-positive gay men. Future research should attend more closely to the role of ethnicity, as well as other factors which our study did not address but which are likely to affect the formation of parenting desires and intentions, such as religion and geographical location.

## Conclusions

Discussing parenthood with gay men diagnosed with HIV was uncommon, according to both patients and healthcare practitioners who took part in our study. As parenting desires and intentions were rarely discussed with men in HIV care, clinicians commonly assumed that having children was of little interest to gay men, while some men questioned if it was possible for them to become parents considering their HIV status. Our findings highlight the potentially beneficial effects of emphasising that having children is a possibility at diagnosis, regardless of patients’ gender or sexuality. Conveying this information seems meaningful, not only to men who want to become parents in the future but also to others, as it appears to alleviate fears about mortality and ill health.

## Supplementary Information

**Additional file 1.**

**Additional file 2.**

## Data Availability

Due to ethical restrictions, data are not publicly available as they contain potentially identifying information of a sensitive nature. Reasonable requests for access should be directed to the corresponding author.

## Footnotes

1 It also echoes narratives of gay fathers who often recall how coming out as gay earlier in their lives had made them assume that they would never have children [16, 20-23].
