# Supplementary material for: Discussing parenthood with gay men diagnosed with HIV: a qualitative study of patient and healthcare practitioner perspectives
Source: BMC Public Health. 2021 Dec 19;21:2300. doi: 10.1186/s12889-021-12285-4 (PMC8684690; doi:10.1186/s12889-021-12285-4)
Supplement: Supplementary file 1 — Additional file 1. [file 12889_2021_12285_MOESM1_ESM.docx]

**Before recording**

Information sheet xx Consent form xx

Water xx Recorder xx

Questions?

Yes xx No xx

…………………………………………………………………………………………………………………………………………………………….

Comments

…………………………………………………………………………………………………………………………………………………………….

**Background information**

Current role

…………………………………………………………………………………………………………………………………………………………….

Previous roles

…………………………………………………………………………………………………………………………………………………………….

Patient demographics (% MSM)

…………………………………………………………………………………………………………………………………………………………….

**Parenthood/reproductive health and HIV care**

Conversations about parenthood in the clinic

…………………………………………………………………………………………………………………………………………………………….

Most common challenges

…………………………………………………………………………………………………………………………………………………………….

Views on service provision

…………………………………………………………………………………………………………………………………………………………….

Views on safe conception

…………………………………………………………………………………………………………………………………………………………….

**Parenthood/reproductive health and MSM**

Conversations about parenthood in the clinic

…………………………………………………………………………………………………………………………………………………………….

Perceptions of parenting desire

…………………………………………………………………………………………………………………………………………………………….

Differences among MSM (sexuality, age, etc.)

…………………………………………………………………………………………………………………………………………………………….

Need for information?

…………………………………………………………………………………………………………………………………………………………….

**Conclusion**

Is there anything you would like to add before we finish?

…………………………………………………………………………………………………………………………………………………………….

**After recording**

Questions?

Yes xx No xx

…………………………………………………………………………………………………………………………………………………………….

Comments

…………………………………………………………………………………………………………………………………………………………….

**INDICATIVE QUESTIONS**

**Background information**

What is your current role?

How long have you worked in HIV and sexual health?

Prior to your current job, did you work at any other clinics or hospitals?

How would you describe the patient population you have contact with in your practice?

What proportion of your patients are men who have sex with men (MSM)?

**Parenthood/reproductive health and HIV care**

How common is it for you to discuss parenthood or reproductive health with your patients?

What are the most common scenarios in which these topics come up?

Who usually initiates these topics – you or the patient?

What are the most common challenges that you face in these conversations?

Can you recall any situations when you found it difficult to offer your patient advice?

To what extent do you think reproductive health is adequately addressed in HIV care?

Is there anything about the current service provision that you would like to see change?

Considering the current state of medical knowledge, how safe is it, in your view, for men living with HIV to conceive children with their own sperm?

**Parenthood/reproductive health and MSM**

Do you ever discuss parenthood or reproductive health with your MSM patients?

Could you give me any examples of situations where you’ve had this kind of discussion?

How common do you think it is for MSM living with HIV to consider having children?

Thinking about your MSM patients, what proportion would you say have sex with women as well as men? What proportion would you say identify as bisexual?

Do you think there are any differences between younger and older MSM in how they approach intimate life?

To what extent would you say it is relevant to discuss reproductive health with MSM?

To what extent do you feel prepared to discuss reproductive health with MSM?

Can you recall any situations when you found it difficult to offer MSM advice regarding reproductive health?

Do you think there is a need for any kind of service or information?

**Conclusion**

Is there anything you would like to add before we finish?

Would you like to elaborate on anything that we have talked about?

Do you have any final comments to make?
