# Supplementary material for: Discussing parenthood with gay men diagnosed with HIV: a qualitative study of patient and healthcare practitioner perspectives
Source: BMC Public Health. 2021 Dec 19;21:2300. doi: 10.1186/s12889-021-12285-4 (PMC8684690; doi:10.1186/s12889-021-12285-4)
Supplement: Supplementary file 2 — Additional file 2. [file 12889_2021_12285_MOESM2_ESM.docx]

**Before recording**

Information sheet xx Consent form xx

Water xx Recorder xx

Questions?

Yes xx No xx

…………………………………………………………………………………………………………………………………………………………….

Comments

…………………………………………………………………………………………………………………………………………………………….

**Introduction**

What made you want to take part in this study?

…………………………………………………………………………………………………………………………………………………………….

**Background information**

Age ………………… Came out ………………… Diagnosed ………………… Started treatment ………………….

…………………………………………………………………………………………………………………………………………………………….

Gay/bisexual/other

…………………………………………………………………………………………………………………………………………………………….

Single/in a relationship

…………………………………………………………………………………………………………………………………………………………….

Country of origin

…………………………………………………………………………………………………………………………………………………………….

Before London

…………………………………………………………………………………………………………………………………………………………….

Ethnicity

…………………………………………………………………………………………………………………………………………………………….

Religion

…………………………………………………………………………………………………………………………………………………………….

Education

…………………………………………………………………………………………………………………………………………………………….

Employment

…………………………………………………………………………………………………………………………………………………………….

HIV diagnosis/treatment

…………………………………………………………………………………………………………………………………………………………….

**Intimate and personal relationships**

***Partners***

Age ………………… Nationality ………………………… Ethnicity ……………………… HIV status …………………

…………………………………………………………………………………………………………………………………………………………….

Current

…………………………………………………………………………………………………………………………………………………………….

Previous

…………………………………………………………………………………………………………………………………………………………….

HIV disclosure

…………………………………………………………………………………………………………………………………………………………….

Conversations about parenthood

…………………………………………………………………………………………………………………………………………………………….

***Family of origin***

Parents

…………………………………………………………………………………………………………………………………………………………….

Siblings

…………………………………………………………………………………………………………………………………………………………….

Coming out

…………………………………………………………………………………………………………………………………………………………….

HIV disclosure

…………………………………………………………………………………………………………………………………………………………….

Conversations about parenthood

…………………………………………………………………………………………………………………………………………………………….

***Friends***

Mostly gay/straight/men/women?

…………………………………………………………………………………………………………………………………………………………….

Parents?

…………………………………………………………………………………………………………………………………………………………….

HIV-positive?

…………………………………………………………………………………………………………………………………………………………….

HIV disclosure

…………………………………………………………………………………………………………………………………………………………….

Conversations about parenthood

…………………………………………………………………………………………………………………………………………………………….

**Feelings about parenthood**

Parenting desire

…………………………………………………………………………………………………………………………………………………………….

Change over time

…………………………………………………………………………………………………………………………………………………………….

Coming out

…………………………………………………………………………………………………………………………………………………………….

HIV diagnosis

…………………………………………………………………………………………………………………………………………………………….

Not having children

…………………………………………………………………………………………………………………………………………………………….

**Pathways to parenthood**

Adoption/fostering

…………………………………………………………………………………………………………………………………………………………….

Surrogacy

…………………………………………………………………………………………………………………………………………………………….

Co-parenting/donating sperm

…………………………………………………………………………………………………………………………………………………………….

Single parenthood

…………………………………………………………………………………………………………………………………………………………….

HIV

…………………………………………………………………………………………………………………………………………………………….

**Parenthood and HIV care**

Conversations about parenthood

…………………………………………………………………………………………………………………………………………………………….

Support outside clinic?

…………………………………………………………………………………………………………………………………………………………….

Need for information?

…………………………………………………………………………………………………………………………………………………………….

**Conclusion**

Is there anything you would like to add before we finish?

…………………………………………………………………………………………………………………………………………………………….

**After recording**

Voucher & Oyster card xx

Consent form xx

Questions?

Yes xx No xx

…………………………………………………………………………………………………………………………………………………………….

Comments

…………………………………………………………………………………………………………………………………………………………….

**INDICATIVE QUESTIONS**

**Introduction**

What made you want to take part in this study?

Do you remember what you thought when you found out about this study?

Why did you think taking part in this study was worthwhile?

**Intimate and personal relationships**

Could you tell me a little more about your current/past relationship(s)?

Have/did you ever talk(ed) about the possibility of having children?

Could I ask you a few questions about your family?

Who do you consider to be your family?

How would you describe your relationship with your family?

Are your family aware that you are gay/bisexual?

Are your family aware that you are living with HIV?

Have you ever talked with your family about the possibility of having or not having children?

Could you tell me a little more about your friendship networks?

How would you describe your friends in terms of gender and sexuality?

Are your friends aware that you are living with HIV?

Do you have any friends who you know are also living with HIV?

How common is parenthood in your friendship circles?

Do you have any parents among your non-heterosexual friends?

How did they become parents?

Is parenthood something that you ever talk about with your friends?

How do you think your non-heterosexual friends feel about having children?

**Feelings about parenthood**

Thinking about the future, would you want to have children at some point in your life?

Has this changed over time or have you always felt about it this way?

Do you remember how you felt about having children when you were younger?

Did your views change in any way when you realised that you were gay/bisexual?

Did your views change in any way when you realised that you were HIV-positive?

Does HIV affect in any way how you feel about parenthood now?

How do you feel about not having children in the future?

**Pathways to parenthood**

I’d like to ask you a little more about the various ways people can become parents…

Have you ever thought about how you could potentially become a parent?

Would you still consider it as a possibility?

What other options would you consider?

I’d like to ask you whether you have any particular views about some other ways to have children…

Have you ever considered adoption/fostering/surrogacy/co-parenting with a female friend?

Is there anything you would think about if you were to consider having children more seriously?

Does HIV affect in any way how you feel about the different ways to have children?

**Parenthood and HIV care**

Have you ever discussed parenthood or reproductive health in the clinic?

Would you have liked to discuss it (more)?

What would be the best way to approach this topic with gay and bisexual men?

How relevant is it, in your view, to discuss parenthood or reproductive health as part of HIV care?

If you were ever planning to have children, how comfortable would you feel seeking advice?

Do you think there is a need for any kind of service or information?

**Conclusion**

Is there anything you would like to add before we finish?

Would you like to elaborate on anything that we have talked about?

Do you have any final comments to make?
